# Clinical aspects of envenomation caused by *Tityus obscurus* (Gervais, 1843) in two distinct regions of Pará state, Brazilian Amazon basin: a prospective case series

**DOI:** 10.1186/1678-9199-20-3

**Published:** 2014-02-11

**Authors:** Pedro PO Pardal, Edna AY Ishikawa, José LF Vieira, Johne S Coelho, Regina CC Dórea, Paulo AM Abati, Mariana MM Quiroga, Hipócrates M Chalkidis

**Affiliations:** 1 Laboratory of Medical Entomology and Venomous Animals, Center of Tropical Medicine, Pará Federal University, Av. Generalíssimo Deodoro 92, Umarizal 66055-240 Belém, Pará state, Brazil; 2 Departament of Biological Science, São Paulo State University (UNESP – Univ Estadual Paulista), Av. Dom Antonio 2100, 19806-390 Assis, São Paulo state, Brazil; 3 Hospital Municipal de Santarém, Av. Presidente Vargas, 1539, Santa Clara, 68005-110 Santarém, Pará state, Brazil; 4 Faculdades Integrada do Tapajós, Rua Rosa Vermelha, 335, Aeroporto Velho, 68010-200 Santarém, Pará state, Brazil

**Keywords:** Scorpionism, *Tityus obscurus*, Envenoming, Neurological symptoms, Brazilian Amazon

## Abstract

**Background:**

Scorpion envenomations are a major public health problem in Brazil, whose most dangerous cases are attributable to the genus *Tityus*. This study was designed to compare the clinical and demographic features of envenomations by *Tityus obscurus* in two areas of the state of Pará located in the Amazon basin.

Were compared demographic findings, local and systemic signs and symptoms of human envenomations caused by *T. obscurus* that occurred in western and eastern areas of the state.

**Results:**

Forty-eight patients with confirmed envenomation by *T. obscurus* were evaluated from January 2008 to July 2011. Most of them came from the eastern region, where male and female patients were present in similar numbers, while males predominated in the west. Median age groups were also similar in both areas. Most scorpion stings took place during the day and occurred significantly more frequently on the upper limbs. The time between the sting and admission to the health center was less than three hours in both areas. Most eastern patients had local manifestations while in the west, systemic manifestations predominated. Local symptoms were similar in both areas, but systemic signs and symptoms were more common in the west. Symptoms frequently observed at the sting site were local and radiating pain, paresthesia, edema, erythema, sweating, piloerection and burning. The systemic manifestations were significantly higher in patients from the west. Futhermore, neurological symptoms such as general paresthesia, ataxia, dysarthria, myoclonus, dysmetria, and electric shock-like sensations throughout the body were reported only by patients from the west.

**Conclusion:**

The present study shows that two regions of Para state differ in the clinical manifestations and severity of confirmed envenomation by *T. obscurus* which suggests a toxicity variation resulting from the diversity of *T. obscurus* venom in different areas of the Brazilian Amazon basin, and that *T. serrulatus* antivenom can be successfully used against *T. obscurus*.

## Background

Scorpion envenomations are a major public health problem in Brazil where approximately 50,000 cases are reported annually. Among the 18 scorpion families described in the world, only four (Bothriuridae, Buthidae, Chactidae and Hemiscorpiidae) have been reported in Brazil [1,2]. There are at least 160 scorpion species in the country of which four (*T. serrulatus*, *T. bahiensis*, *T. stigmurus* and *T. obscurus*) are of medical relevance [3,4]. Among the 44 scorpion species that occur in the Brazilian Amazon region, only *T. metuendus*, *T. silvestris* and *T. obscures* were responsible for human envenomations [2,4].

*Tityus obscurus* (Gervais, 1843) (Scorpiones: Buthidae) is a senior synonym of *T. paraensis* Kraepelin, 1896 and *T. cambridgei* Pocock, 1897 [5]. Furthermore, it is widely distributed in the Brazilian Amazon, notably in Mato Grosso, Pará and Amapá states and has been the most important species responsible for scorpion stings in those areas [3,4,6,7].

The neurotoxic effect of scorpion species within the Buthidae family has been reported in several countries [8,9]. In Brazil, local envenoming causes pain, erythema, and swelling. Systemic envenoming usually causes vomiting, sweating, hypersalivation, priapism, bradycardia or tachycardia, arterial hypotension or arterial hypertension, and cardiac failure, with little or no effect on the central nervous system [10,11]. There are few data describing the clinical and epidemiological features of envenomations due to *T. obscurus* in the Brazilian Amazon, in particular the difference in the occurrence of neurological signs or symptoms in some previously surveyed areas [7]. Therefore, this study was designed to compare the clinical and demographic features of envenomations by *T. obscurus* in two areas of the Amazon basin located in the state of Pará.

## Methods

### Study area

**Figure 1:**
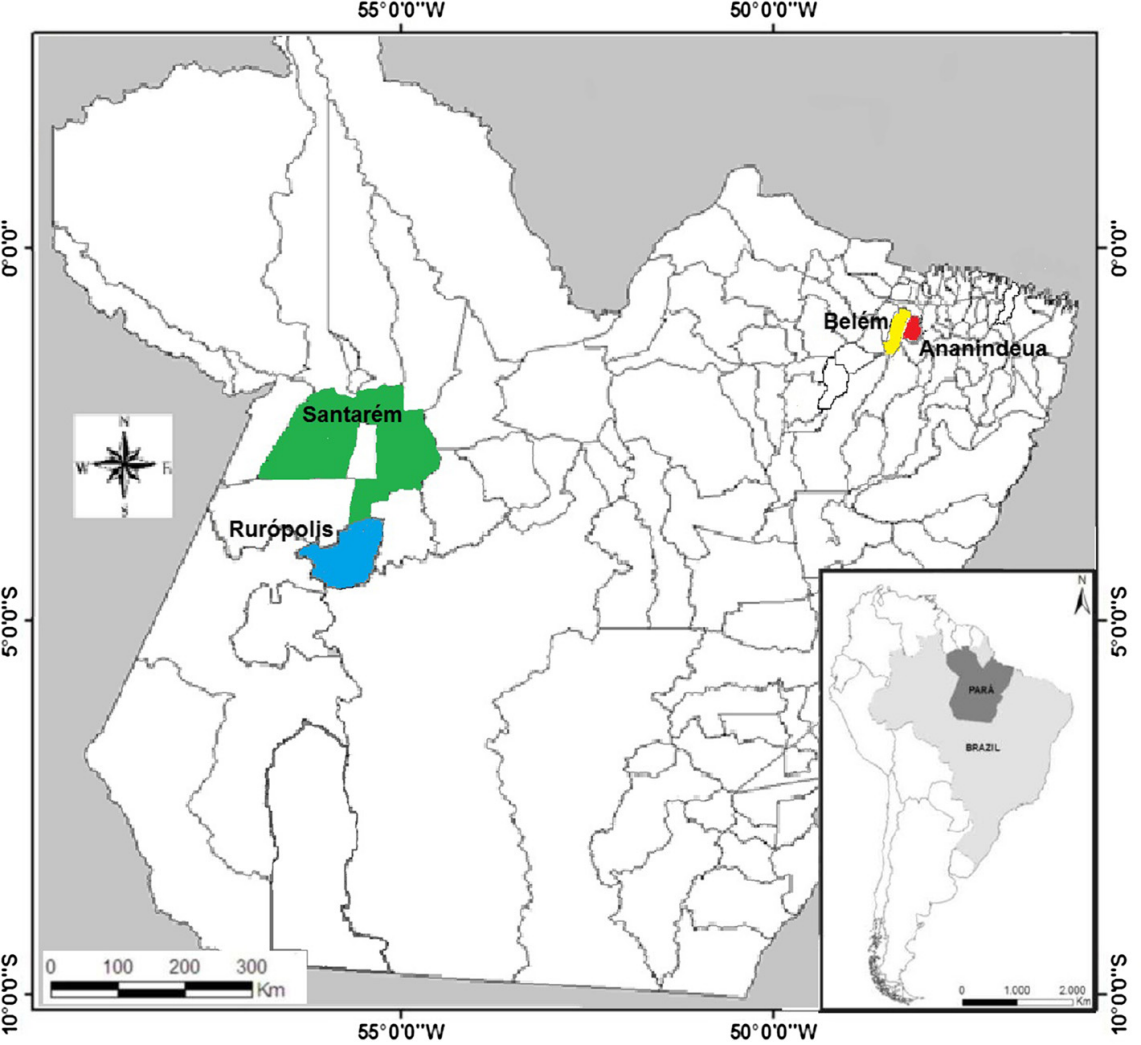
Study areas in Pará state, northern Brazil.

This prospective study was carried out from January 2008 to July 2011. Four municipalities were chosen: Belém and Ananindeua in the east, and Santarém and Rurópolis in western Pará state, located in the eastern Brazilian Amazon, encompassing an area of about 850 km^2^ (Figure 1). Belém is the capital of the state with an area of 1,059.402 km^2^ and 1,393,399 inhabitants. Ananindeua is located in the metropolitan region of Belém, with an area of 190.452 km^2^ and 471,980 inhabitants. Santarém and Rurópolis are typical rural municipalities. The former is located at the edge of the Amazon River with an area of 22,886.761 km^2^ and 294,580 inhabitants. Rurópolis has 7,021.305 km^2^ and 40,078 inhabitants. The four cities are surrounded by tropical forests. The climate in these areas is hot and humid with an average annual temperature ranging between 22°C and 34°C.

### Patients

**Figure 2:**
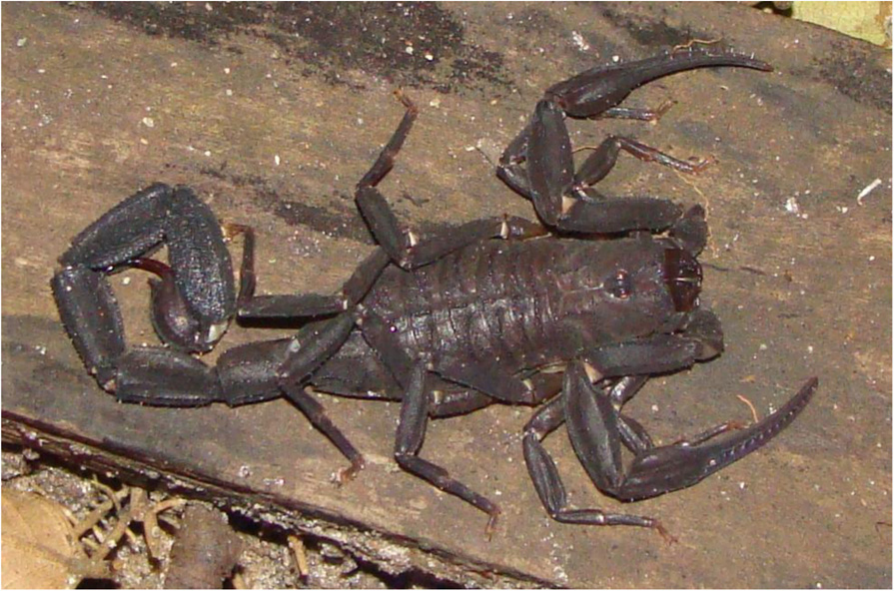
**Female specimen of *Tityus obscurus *(Gervais, 1843) with length of 10 cm from the eastern region of Pará state, Brazil.** It had been provided by a patient who was showing systemic manifestations upon hospital admission.

Patients with proven envenomations by *T. obscurus* but no previous history of neurological disease were included in the study. Initially, patients were attended in the emergency rooms of public hospitals of the selected cities. Altogether 48 patients with confirmed envenomation by *T. obscurus* were evaluated: 34 patients from eastern Pará state and 14 patients from the western part. Participants were admitted on a voluntary basis and received follow-up based on the identification of the specimens provided to the medical staff as *T. obscurus* (Gervais, 1843) (Figure 2).

The specimens were provided voluntarily by the patients upon hospital admission. They were initially identified at the Medical Entomology Laboratory of the Tropical Medicine Centre at the Pará Federal University according to the description of Lourenço and Leguin [5] and later confirmed by the Arthropods Laboratory at Butantan Institute, São Paulo, Brazil. All specimens were subsequently preserved in 70% ethanol and deposited at Pará Federal University.

Clinical and demographic data from each patient were recorded, including age, gender, sting site, time elapsed from the accident to initial medical attention, as well as the severity of poisoning and treatment at admission. Biochemical and hematological evaluations were not performed. The patients were allocated into one of the following four classes according to the recommendations of the Scorpion Consensus Expert Group [12]:

• Level 0 (dry sting or asymptomatic).

• Level 1 (local manifestations): pain, burning sensation, erythema, paresthesia, swelling and tingling.

• Level 2 (minor systemic manifestations): agitation, headache, nausea, vomiting, sweating, unhealthy pallor, salivation, somnolence/lethargy, tachycardia, hypertension, hypothermia, hyperthermia, myoclonia, fasciculation, ataxia, dystonia, miosis and mydriasis.

• Level 3 (major systemic manifestations): hypotension, ventricular arrhythmia, bradycardia, cardiovascular collapse, cyanosis, dyspnea, pulmonary edema, paralysis and Glasgow score ≤6 (in absence of sedation).

All patients with systemic manifestations were treated with scorpion antivenom (5 mL/vial; 1 mL neutralizes 1 mg of *T. serrulatus* reference venom in mice), corresponding to F(ab’)2 polyspecific hyperimmune equine antivenom raised against a venom from *T. serrulatus* produced by Ezequiel Dias Foundation, Minas Gerais state, Brazil.

### Statistical analysis

Data are expressed as the median and the 25th and 75th percentiles [IQR, interquartile range]. Pearson’s chi-square method and the Fischer exact test were employed to compare variables between groups. Statistical analyses were performed using the software package Statistica® (Version 6, Stat Soft 2001, USA). All *p* values were two-tailed, and p < 0.05 was considered statistically significant.

### Ethical aspects

Informed consent was obtained from each patient or from a parent thereof. This study was approved by the Ethics Committee of the Tropical Medicine Center of Pará Federal University, document number 038/07. The care of the scorpion specimens was authorized by the Brazilian Environmental Institute (IBAMA), document number 11727–2.

## Results

Most of the patients with confirmed envenomations were living in the eastern portion of Pará state (n = 34, 71%), which presented an equal distribution of males and females whereas in the west males predominated. The ages of patients were similar in both areas with a median of 31.5 (23–41) years in the east, and 31.5 (20–50) years in the west, but the accidents happened mainly to patients older than 15 in both areas. The stings occurred more frequently in upper rather than lower limbs or other parts of the body in both regions. The majority of scorpion stings occurred during the day in both areas. The interval between the sting and hospital admission was less than three hours in both regions, with a median of 1.7 (0.3-2.0) hours in the east versus 1.3 (1.0-3.0) hours in the west (Table 1).

**Table 1:** **Demographic findings of patients envenomed by *Tityus obscurus *** **(Gervais, 1843) in two study areas of Pará state, Brazil**

**Parameters**	**Eastern (n = 34)**	**Western (n = 14)**	***p *** **value**
**Gender**			0.52
Male	17	9	
Female	17	5	
**Age (years)**			0.73
≤ 5	1	1	
6-14	4	1	
≥ 15	29	12	
*Md (IQR*)*	31.5 (23–41)	31.5 (20–50)	
**Site of sting**			0.65
Upper limbs	24	8	
Lower limbs	8	5	
Other site	2	1	
**Time of sting**			0.02
7 a.m. to 6 p.m.	25	10	
7 p.m. to 12 a.m.	9	4	
**Time elapsed until admission (hours)**			0.83
< 1	22	8	
2-3	5	2	
3 ≥	7	4	
*Md* (*IQR*)*	1.0 (0.3-2.0)	1.3 (1.0-3.0)	

*Median and interquartile range.

The local manifestations were similar in both regions, but the presence of systemic manifestations predominated in the west. Antivenom (p = 0.005) was used mainly in patients from the western region (Table 2). It was also observed that patients who were 15 or older had a higher occurrence of systemic manifestations in the west (eight patients) and in the east (five patients), respectively (data not shown).

**Table 2:** **Clinical findings of patients envenomed by *Tityus obscurus *** **(Gervais, 1843) in two study areas of Pará state, Brazil**

**Parameters**	**Eastern (n = 34)**	**Western (n = 14)**	***p *** **value**
Severity of symptoms*			
Dry sting	2	0	
Local manifestations	32	14	
Systemic manifestations	6	9	
Signs and symptoms*			0.005
Level 1	26	5	
Level 2	6	9	
**Treatment**			0.005
Painkillers	26	5	
Antivenom	6	9	

*According to the International the Scorpion Consensus Expert Group [12].

The signs and symptoms frequently observed at the sting site were local and radiating pain, paresthesia, edema, erythema, sweating, piloerection and burning (Table 3). The paresthesia and radiating pain predominated in patients from the western region. In addition, burning and piloerection were reported only in these patients.

**Table 3:** **Local and systemic signs and symptoms of patients envenomed by *Tityus obscurus *** **(Gervais, 1843) in two study areas of Pará state, Brazil**

Parameters*	**Eastern (n = 34)**	**Western (n = 14)**
**Local manifestations**		
Pain	30	14
Pain radiating	2	9
Paresthesia	16	12
Edema	12	7
Erythema	14	5
Sweating	1	3
Piloerection	0	4
Burning	0	1
**Systemic manifestations General:**		
Sweating	2	5
Headache	1	0
Agitation	0	4
Tremors	0	5
Prostration	0	1
Asthenia	0	2
Chills	0	1
**Gastrointestinal**		
Nausea	3	4
Abdominal cramps	0	3
Vomiting	0	2
Sialorrhea	0	1
**Cardiorespiratory**		
Hypertension	0	1
Tachypnea	1	0
**Ophthalmologic**		
Blurred vision	1	5
Conjunctival hyperemia	0	4
Photophobia	0	1
**Neurological**		
Somnolence	1	4
Dizziness	3	0
Confusion	1	0
Electric shock-like sensations	0	7
Myoclonus	0	9
Ataxia	0	4
Paresthesia	0	5
Dysarthria	0	6
Dysmetria	0	3
Fasciculation	0	3
Motor incoordination	1	1

*Some patients had more than one sign or symptom of envenoming.

In both regions, all patients showed minor systemic manifestations (Table 3). The general, ophthalmological and neurological signs and symptoms were more frequent in patients from the western region. The most frequent findings in eastern patients were nausea, dizziness and sweating, whereas those from the west more often reported blurred vision, tremors, sweating, agitation, nausea and somnolence. Some neurological signs and symptoms such as myoclonus, electric shock-like sensations in the body, dysarthria, paresthesia, ataxia and dysmetria were reported only in patients from the western region. All patients evolved to healing after treatment. Patients with only local manifestations were treated with analgesics and discharged from the hospital between 6 to 12 hours after admission. On the other hand, patients with local and systemic manifestations were treated as follows: they had their vital functions monitored and received supportive care; benzodiazepines were administered to those with myoclonus; and each patient received a dosage of 15 mL of scorpion antivenom which was infused intravenously over a 20-minute period. After antivenom administration, no adverse effects were ever noted in patients from both regions. Patients from eastern Pará were released from the hospital within 24 hours, whereas in the western region seven patients and five patients were released, respectively, within 48 hours and 72 hours (data not shown).

## Discussion

The majority of patients included in this study were from the eastern region of Pará state, which can be associated with both a higher population density and easier access to medical assistance in the metropolitan area of Belém. Several species of the genus *Tityus* have been described and an annual incidence of 82.4 stings per 100,000 inhabitants was found statewide [2]. However, the range of incidence varied from 6.4 stings per 100,000 inhabitants to 264.4 stings per 100,000 inhabitants, respectively for the east (Belém and Ananindeua) and west (Santarém and Rurópolis).

Hospital admission was found to be delayed in the western region, probably due to difficulties in transportation to a hospital and the existence of few emergency health centers in rural areas of the Amazon region. The increase in the elapsed time between stings and medical assistance may be responsible for the worsening of signs and symptoms of envenomations, thus contributing to the greater occurrence of systemic manifestations in the western region. A previous report showed that clinical signs and symptoms of envenomation worsened when the elapsed time between sting and first medical attendance was above 30 minutes [8].

The finding of similar results in both genders in the study areas corroborated previous reports of envenomations by several scorpion species in Brazil [7,13]. However, in males the frequency of envenomations by *T. obscurus* was slightly higher than had been previously reported for the municipality of Santarém, a finding probably associated with the type of work performed in the rural environment of the western region [7]. Similar distributions of cases amongst various age groups were previously reported in Brazil, Colombia and Turkey [9,13,14].

The high frequency of stings in the upper limbs and the fact that most stings occurred during the day, despite the nocturnal disposition of *T. obscurus*, suggested that this behavior was a defense strategy of the scorpion against a disturbance in its natural habitat [7]. Other reports on *Tityus* species showed a high frequency of stings in the lower limbs, such as those caused by *T. trivittatus* in Argentina [15]. Furthermore, nighttime stings were previously reported in Colombia originating from *T. fuhrmanni*, in South Africa from *Parabuthus granulatus* and in Iran from *Hemiscorpius lepturus*[16-18]. In Brazil, stings by *T. bahiensis* and *T. serrulatus* were reported as having occurred either at night or during the day [11,19].

The frequency of local signs and symptoms was similar in patients from both regions. The most frequently observed manifestations were pain followed by paresthesia, edema and erythema, thus corroborating previous clinical findings on envenomations by *T. obscurus* and *T. silvestris* in the Brazilian Amazon and by *T. stigmurus* in northeastern Brazil, *T. serrulatus* and *T. bahiensis* in southeastern Brazil and other countries [6-8,10,13,17,20]. In Tunisia, envenomations by *H. lepturus* were not accompanied by local inflammatory process, but in Iran local necrosis was reported [21,22].

The severity of envenomations differed between the two regions in that western patients presented higher frequencies of systemic signs and symptoms with an emphasis on the neurological findings. All the patients of this study were included in level 2 of the Scorpion Consensus Expert Group [12], corroborating the finding of Nishikawa *et al*. [23] who have shown that the venom of *T. obscurus* was slightly toxic when compared with venoms of other Brazilian scorpions, such as *T. serrulatus*, *T. bahiensis* and *T. estigmurus*.

Several signs and symptoms have been reported in scorpion envenomations, mostly involving both sympathetic and parasympathetic stimulation as well as central manifestations. A variety of central nervous system (CNS) symptoms has been reported in human scorpionism by *T. serrulatus*, *T. bahiensis* and *T. stigmurus*, including somnolence, dizziness, contracture, tremors and seizures [10,11,20]. However, some neurological findings were observed only in envenomations attributed to *T. obscurus* in the western area and were not found in other regions of Pará state [6]. This was the case in regards to myoclonus, electric shock-like sensations in the body, dysarthria, paresthesia, ataxia and dysmetria. Previous reports of similar clinical manifestations have been attributed to this species in the municipalities of Santarém, Belterra and Prainha in the west of Pará, Brazil and French Guiana, as well as by *Centruroides infamatus* and *C. limpidus tecomanus* in Mexico, *P. granulatus* and *P. transvaalicus* in South Africa and in Zimbabwe [7,8,24,25].

The difference in neurological signs and symptoms caused by *T. obscurus* from eastern and western regions suggests a variability in venom composition. The venom of *T. obscurus* contains at least 102 distinct peptide components, but the complete primary structures of only 18 peptides are known. Three of them are K^+^-channel-specific toxins and the other 15 toxins act on Na^+^-channels [26-28]. The diversity of components in scorpion venom resulting from geographical differences and environmental changes in the habitat has been described for several scorpion species. According to Ruiming *et al*. [29] and Ozkan and Ciftci [30], geographical separations can lead to diversity of components among scorpion venoms. Other authors such as Badhe*et al*. [31] found intraspecific variation between venoms of *Mesobuthus tamulus* from different regions of India; Abdel-Rahman *et al*. [32] observed that a number of factors such as environmental, genetic and geographic ones contributed to intraspecific variation of the toxin from *Scorpio maurus palmatus* populations of different regions of Egypt. In Brazil the variation and diversity in the toxicity from the venom of *T. serrulatus* and other *Tityus* species of medical importance have already been shown [20,33].

The specific treatment for scorpion envenomations in Brazil is based on the administration of species-specific antivenom, obtained from plasma of horses immunized with the venoms of *T. serrulatus*, which has been used effectively for the treatment of human scorpionism attributable to *T. serrulatus*, and other species such as *T. stigmurus* and *T. bahiensis*[3,10,11,20]. In addition, an *in vivo* and *in vitro* study showed that the antivenom of *T. serrulatus* can also be used successfully against *T. obscurus*[23].

## Conclusion

The present study showed that cases of confirmed envenomation by *T. obscurus* in two regions of the state of Pará, Brazil presented different clinical manifestations and severity. The neurological manifestations such as myoclonus, dysarthria, electric shock-like sensations throughout the body, ataxia and dysmetria, were reported only by patients from the western portion of the state, which suggests variation and diversity in the toxicity of *T. obscurus* venom from different areas of the Brazilian Amazon basin, and that *T. serrulatus* antivenom can be successfully used against *T. obscurus*.

### Ethics committee approval

The present study was approved by the Ethics Committee of the Tropical Medicine Center of Pará Federal University, document number 038/07. The care of the scorpion specimens was authorized by the Brazilian Environmental Institute (IBAMA), document number 11727–2. In addition, informed consent was obtained from each patient or from a parent thereof.

## Competing interests

The authors declare that there are no competing interests.

## Authors’ contributions

PPOP planned the study, participated in its design, coordination and helped to draft the manuscript. EAYI, JLFV, HMC and RCCD participated in the study design, performed the statistical analysis and provided technical support and scientific discussions. JSC identified the scorpion specimens provided by the patients. MMMQ and PAMA were physicians in charge of the patients. All authors read and approved the final manuscript.
